# The Effects of Lifestyle on the Risk of Lyme Disease in the United States: Evaluation of Market Segmentation Systems in Prevention and Control Strategies

**DOI:** 10.3390/ijerph182412883

**Published:** 2021-12-07

**Authors:** Esra Ozdenerol, Rebecca Michelle Bingham-Byrne, Jacob Daniel Seboly

**Affiliations:** 1 Spatial Analysis and Geographic Education Laboratory, Department of Earth Sciences, University of Memphis, Memphis, TN 38152, USA; rmbngham@memphis.edu; 2 Department of Geosciences, Mississippi State University, Starkville, MS 39762, USA; jds1565@msstate.edu

**Keywords:** geographic information systems, lifestyle segment, LifeModes, market segmentation, market intelligence, tick-borne diseases, Lyme disease incidence, risk mapping

## Abstract

The aim of this study was to investigate lifestyles at risk of Lyme disease, and to geographically identify target populations/households at risk based on their lifestyle preferences. When coupled with geographically identified patient health information (e.g., incidence, diagnostics), lifestyle data provide a more solid base of information for directing public health objectives in minimizing the risk of Lyme disease and targeting populations with Lyme-disease-associated lifestyles. We used an ESRI Tapestry segmentation system that classifies U.S. neighborhoods into 67 unique segments based on their demographic and socioeconomic characteristics. These 67 segments are grouped within 14 larger “LifeModes” that have commonalities based on lifestyle and life stage. Our dataset contains variables denoting the dominant Tapestry segments within each U.S. county, along with annual Lyme disease incidence rates from 2000 through 2017, and the average incidence over these 18 years. K-means clustering was used to cluster counties based on yearly incidence rates for the years 2000–2017. We used analysis of variance (ANOVA) statistical testing to determine the association between Lyme disease incidence and LifeModes. We further determined that the LifeModes Affluent Estates, Upscale Avenues, GenXurban, and Cozy Country Living were associated with higher Lyme disease risk based on the results of analysis of means (ANOM) and Tukey’s post hoc test, indicating that one of these LifeModes is the LifeMode with the greatest Lyme disease incidence rate. We further conducted trait analysis of the high-risk LifeModes to see which traits were related to higher Lyme disease incidence. Due to the extreme regional nature of Lyme disease incidence, we carried out our national-level analysis at the regional level. Significant differences were detected in incidence rates and LifeModes in individual regions. We mapped Lyme disease incidence with associated LifeModes in the Northeast, Southeast, Midcontinent, Rocky Mountain, and Southwest regions to reflect the location-dependent nature of the relationship between lifestyle and Lyme disease.

## 1. Introduction

The ability to profile a target audience in terms of its morbidity (i.e., sickness) characteristics and its health service needs is becoming increasingly critical for successful marketing initiatives [1]. A wide variety of healthcare entities—whether providers of care, producers of medical supplies and drugs, or organizations providing goods or services to the healthcare industry—are required to market themselves to their prospective customers.

To understand prospective customers, the basic questions start with who gets sick, what they get sick from, and where they get sick. Since morbid conditions are not randomly distributed within the population, but are concentrated within certain segments of it, this baseline information can be expanded by segmenting the population based on the clustering of lifestyle attributes—consumer behaviors, exercise patterns, recreational activities, dietary preferences, and so forth. Borrowing market intelligence tools such as market segmentation (i.e., geodemographic segmentation, life style segments), we can identify a population, determine its lifestyle clusters, and estimate its propensity for various conditions. Market segmentation is a common marketing strategy that involves grouping potential customers into lifestyle segments by households, zip codes, and block groups [2]. This granular information could be used to plan health initiatives, develop treatment modalities, improve the delivery of care, and develop marketing programs for healthcare organizations, effective health insurance plans (private and public), and cost-effective approaches to the provision of care.

As the impact of mortality on the population is declining, the shift from acute conditions seemingly affecting populations at random to the growth of lifestyle-generated chronic conditions also serves to increase this interest in investigating the correlation between morbidity patterns and lifestyle segments [1]. Chronic diseases are much more selective in their impacts, resulting in lifestyle-related disparities in health status [3]. This effort involves the identification of and attention to the non-medical efforts that influence health status and the social contributors to ill health—factors clearly addressed through lifestyle-oriented morbidity analysis. To investigate this, we chose the most common infectious disease and chronic condition in North America: Lyme disease. The comprehensive, timely, and detailed data on Lyme disease morbidity levels provided by the CDC helped us to demonstrate the impact of lifestyle on Lyme disease. Moreover, North America is likely to dominate the global Lyme disease therapeutics market, due to the high rate of incidence of Lyme disease and high awareness about and adoption of new diagnostic methods [4]. This paper offers a unique human social behavioral approach to effectively analyze the impact of Lyme disease on American households by their lifestyle characteristics. We specifically tested four research questions: “Is there a difference in average Lyme disease incidence rates among different LifeModes?”, “Which LifeModes have incidence rates that are higher/lower than average?”, “Which pairs of LifeModes have significantly different incidence rates?”, and “Which LifeModes could potentially have the maximum incidence rate?” We focused on comparing each LifeMode’s mean to the national mean in order to ascertain spatial and temporal patterns of high-risk households and the effects of lifestyle on the risk of Lyme disease infection in the United States.

Our approach provides actionable information for key stakeholders with respect to the focus of interventions and the implementation of prevention and control policies to these specific households exhibiting spatial and temporal patterns of high risk of Lyme disease.

## 2. Human Social Behaviors Affecting the Risk of Contracting Lyme Disease

Lyme disease has become one of the most prevalent tick-borne diseases in the United States [5]. Lyme disease most commonly occurs in the upper Midwest and Northeastern United States but, over time, cases are starting to emerge in other areas, including California, contracted via *Ixodes pacificus* [6]. Much of this is due to various climate factors allowing for larger tick niches. Even though environmental and climatic factors are driving the increase in the Lyme disease vectors in these emerging areas, human social behaviors also affect the risk of contracting Lyme disease.

Recent studies of human social behavior researched the links between human activity, mobility patterns, and tick exposure in Lyme-disease-endemic areas of the United States and new emerging areas in Canada [7,8,9,10]. They used mechanisms of collecting data on human behavior (e.g., retrospective questionnaires) as well as smartphone applications to understand human behaviors affecting tick exposure. Moreover, they engaged the general public in active tick prevention and reporting in different regions of the United States and Canada. Bouchard et al. integrated social behavioral and Lyme disease risk maps in the Montérégie region of southern Quebec, Canada, where Lyme disease is a newly endemic disease [7]. Spatial variation in Lyme disease knowledge, risk perceptions, and related behaviors in the population were measured using web survey data collected in 2012. These data were used as a proxy for the social-behavioral component of risk. Tick vector population densities were measured in the environment during field surveillance from 2007 to 2012 to provide an index of the ecological component of risk. Social-behavioral and ecological components of risk were combined with human population density to create integrated risk maps. Map predictions were validated by testing the association between high-risk areas and the current spatial distribution of human Lyme disease cases. This study demonstrated that social survey data are a valuable but underutilized source of information for understanding regional variation in Lyme disease exposure, and for integrating this information into risk maps.

Fernandez at al. designed the Tick App as a survey tool to collect data on human behavior and movements associated with tick exposure, while raising awareness among the general public by engaging app users in tick identification and reporting [8]. The Tick App consists of an enrollment survey to identify general risk factors, daily surveys to collect data on human activities and tick encounters as “Tick Diaries”, a survey to enter the details of tick encounters coupled with tick identification services as “Report a Tick”, and educational materials. They found that most users owned a pet, frequently engaged in outdoor activities (occupational, recreational, and/or peridomestic), and lived in the Midwest and Northeast regions. These factors increased significantly in counties with high Lyme disease incidence, or with a recent increase in the number of reported cases in low-incidence counties. Recurring users had a similar demographic profile to all users, but participated in outdoor activities more frequently. The number of active users peaked in June and July, with *Ixodes scapularis* nymphal activity peaking from late May through July. The number of “Tick Diaries” submitted per user was higher for older age groups and in the Midwest, while the number of tick reports increased with the frequency of outdoor activities. This assessment allowed the authors to identify what fraction of the population used the Tick App to tailor the design of potential future tick prevention interventions to the users’ characteristics.

Ozdenerol et al., in their review, concluded that more research needed to be done on activity-based risk, perceptions of risk and known factors, and their influence on individuals’ choices to engage in protective behavior [9]. Donohoe et al. studied the impact of LD on the tourism industry, and found that the tourism industry needs to be considered in terms of employee health, travel choices, and the economic sustainability of tourism in LD-endemic areas [10]. A Czech study by Zeman and Benes [11] found that the liberalized housing in peri-urban locations and the real estate market after political and economic transformations influenced the amount of time people spent outdoors around their homes, which has increased due to lifestyle changes. This process has led to increased contact between the populations and the tick habitats. Linard et al. studied the spatial distribution of LD in Belgium [12]; their findings revealed that LD is associated with recreational and peridomestic outdoor activities in high-income peri-urban areas with isolated houses and forests.

The causal explanation of LD trends was also examined by studies examining the behavioral risk of exposure to tick-borne diseases, focusing on regions where LD is endemic, as well as individuals with occupational exposure [13,14,15]. McKenna et al. evaluated factors motivating high-risk individuals to implement Lyme disease prevention behaviors [13]. Patients presenting to the Lyme Disease Diagnostic Center in New York State completed a voluntary, anonymous questionnaire. Participants who reported having had Lyme disease in the past or having a family member or close friend with Lyme disease were more likely to use preventive behaviors. Increasing age was associated with increased use of preventive behaviors only for participants without a history of Lyme disease. These findings provided information that was important in developing community prevention programs for Lyme disease. They suggested that younger persons without a history of Lyme disease should be targeted for programs that would educate them about Lyme disease. Schwartz et al. conducted a statewide cross-sectional study of risk factors for seropositivity for antibodies against *Borrelia burgdorferi* in outdoor workers in New Jersey; their analyses revealed that any use of insect repellent or antibiotics may have decreased the risk of Lyme disease in these workers; they concluded that Lyme disease is a hazard of outdoor work, and that increased recognition of this fact will be necessary in order to prevent Lyme disease in these workers [14].

Schwartz et al. conducted a second cross-sectional study of outdoor workers (*n* = 758) at high risk of Lyme disease. A questionnaire was administered, and antibodies against *Borrelia* *burgdorferi* and tick salivary gland proteins (anti-tick saliva antibody, a biological marker of tick exposure) were assayed via enzyme-linked immunosorbent assay. The statewide Lyme disease seroprevalence increased from 8.1% in 1988 to 18.7% in 1990. Anti-tick saliva antibody seropositivity varied by county, and was associated with measures of self-reported tick exposure. The data suggested that the prevalence of *B. burgdorferi* infection increased in New Jersey outdoor workers from 1988 to 1990 [15].

Bayles et al. measured the preventive behaviors of visitors to recreational parks in the St. Louis, MO area—an endemic area for tick-borne diseases other than LD [16]. They used geographic stratification techniques, creating 5 km radius buffers around the perimeter of each site, and overlaid the buffers on a map of census blocks with population estimates from the 2010 U.S. census. Based on human population densities, they classified parks as either suburban, exurban, or rural. Results presented significant differences in behaviors across parks. Those in exurban parks were more likely to perform frequent tick checks and use insect repellents, while those in suburban parks were more likely to avoid tick habitats. On the other hand, those in rural parks were less likely to avoid tick habitats.

In this paper, we determined the distribution of Lyme disease based on the geographic distribution of households whose lifestyle segments were identified as having a high propensity for Lyme disease. By identifying target populations at risk based on lifestyle preferences, we could target specific types of households and their locations for epidemiological analysis. In addition, we sought to determine whether the relationship between lifestyle and Lyme disease was location-dependent, meaning that lifestyle-related attributes might contribute to the likelihood of infection when the environmental conditions such as climate, tick and pathogen species range, and tick and pathogen habitat are met. This analysis also led to clues as to human behaviors and travel patterns that affect the risk of contracting Lyme disease, and provided evidence that human social behaviors—such as lifestyle preferences—must also be included in Lyme disease risk maps, even though environmental conditions (e.g., tick habitats, endemic areas) are not met. For zoonotic diseases, researchers have worked with GISs to create surveillance databases to improve the effectiveness of oral vaccine deployment programs by creating risk assessment maps to prioritize areas in which to distribute the oral vaccine to the wildlife [17]. It should be possible to determine whether a community should be targeted for an oral vaccine deployment program to eradicate Lyme disease based on high-risk lifestyle clusters.

Given that virtually every household in the U.S. has been assigned a lifestyle segment, linking segments to geographically identified patients (e.g., Lyme disease incidence) could subsequently predict the demand for health services. For example, at-risk populations in high-risk lifestyle clusters (e.g., segments) can be recruited in clinical trials for human vaccines. Establishing this link can also allow for more efficient, more targeted, and more cost-effective healthcare for Lyme disease. Ozdenerol et al. demonstrated this with respect to COVID-19 and lifestyle characteristics associated with COVID-19 infection and mortality rates at the U.S. county level, and sequentially mapped the impact of COVID-19 on different lifestyle segments. [1]. Moreover, we can also prioritize high-risk lifestyle segments whose lifestyle traits (e.g., travelers) are risky for Lyme disease in non-endemic areas for prevention strategies. Ozdenerol’s methodology [1] aims to be a prototype for converting information on lifestyles into the incidence and prevalence of health conditions (e.g., Lyme disease morbidity) and into the demand for health services and prevention strategies.

## 3. Materials and Methods

### 3.1. Data

We combined data from multiple sources and merged them in geographic information systems (GISs) to create a visual representation through maps. We used the ESRI Tapestry segmentation system [2] to associate lifestyle clusters with Lyme disease. We explicitly describe both ESRI Tapestry segmentation and Lyme disease datasets under separate headings below.

#### 3.1.1. ESRI Tapestry Segmentation System

We used the ESRI Tapestry segmentation system [2], which is available on an annual basis, as population and household counts by Tapestry segment are updated each year. The GIS that supports the ESRI Tapestry segmentation platform enables Experian’s ConsumerView database [18], the Survey of the American Consumer from GfK MRI [19], and the U.S. Census American Community Survey [20] datasets to be brought together as maps to create a complete picture of local communities and neighborhoods across the U.S.

The ESRI Tapestry segmentation system utilizes Experian’s consumer survey, which applies traditional customer profiling techniques such as relationships between purchased products and consumers’ beliefs and life patterns [21,22,23]. When composing lifestyle segments, geographic data represent where the focal groups are located and where they are buying and using products. Behavioral data focus on when the groups are more likely to buy, under what circumstances they would buy, and how they would choose to consume or use the product. Demographics represent the races, gender, age groups, and marital status of customers/consumers. Psychographic data concentrate on their uniqueness, personal preferences and lifestyle choices, what they do in their spare time, what products they chose to free up more spare time, and how they see themselves and their communities, as well as identifying careers, opinions, and income parameters [24,25,26].

ESRI Tapestry segmentation classifies U.S. neighborhoods into 67 unique market segments, based on socioeconomic and demographic factors, and then consolidates these 67 segments into 14 LifeModes with names such as “High Society”, “Senior Styles”, and “Factories and Farms” that have commonalities based on lifestyle and life stages [2]. ESRI Tapestry segmentation data were downloaded from ESRI [26]. Our dataset contains a variable denoting the dominant tapestry segment within each U.S. county. Appendix A shows a description of the traits of the LifeModes in a table.

#### 3.1.2. Lyme Disease Incidence Rates

A dataset downloaded from the CDC Wonder database contains Lyme disease incidence rates from 2000–2017 on a county-by-county basis [27]. County-level population estimates were downloaded from the Census Bureau website in two different datasets: the “Intercensal Estimates of Resident Population for Counties and States: 1 April 2000 to 1 July 2010” and the “County Population Totals and Components of Change: 2010–2017” [28].

Our dataset contains variables denoting the dominant Tapestry segment within each U.S. county and the annual Lyme disease incidence rate from 2000 through 2017, as well as the average incidence over these 18 years. K-means clustering was used to cluster counties based on yearly incidence rates for the years 2000–2017 [29]. The incidence rates per 100,000 people were then calculated using Equation (1):

(1) $$\frac{new\;case\;counts\;per\;county}{\frac{population\;per\;county}{100,000}}$$

**Figure 1:**
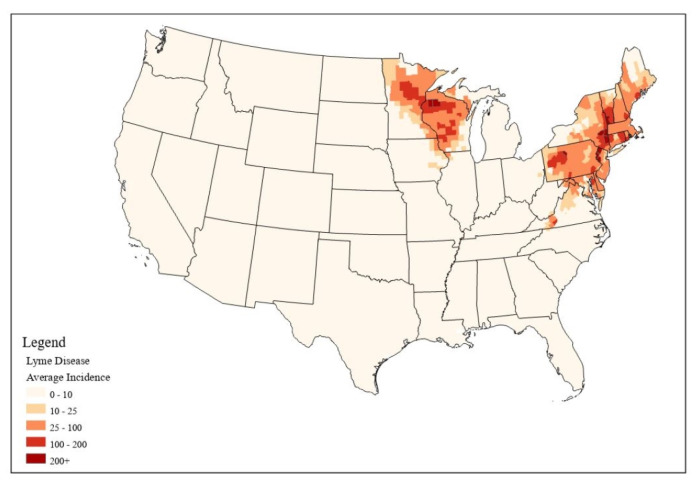
Lyme disease incidence rates in the United States for 2000–2017.

Figure 1 shows Lyme disease incidence rates at the county level in the United States for the period of 2000–2017. The mean incidence rate for all U.S. counties was 8.03 per 100,000 (*n* = 3141). The distribution of incidence rates was extremely right-skewed, with the majority of counties experiencing incidence rates below 1 per 100,000, but some counties experiencing much higher incidence rates. The maximum incidence rate of 641.17 cases per 100,000 was found in Columbia County, New York. Two distinct areas with high Lyme disease risk were evident: one in the Northeastern United States—especially New York, Pennsylvania, and Connecticut—and one in the Midwestern states of Minnesota and Wisconsin. Most counties outside of these areas experienced much lower rates, below 10 per 100,000. All numbers represent cases per 100,000 people.

### 3.2. Methods

**Figure 2:**
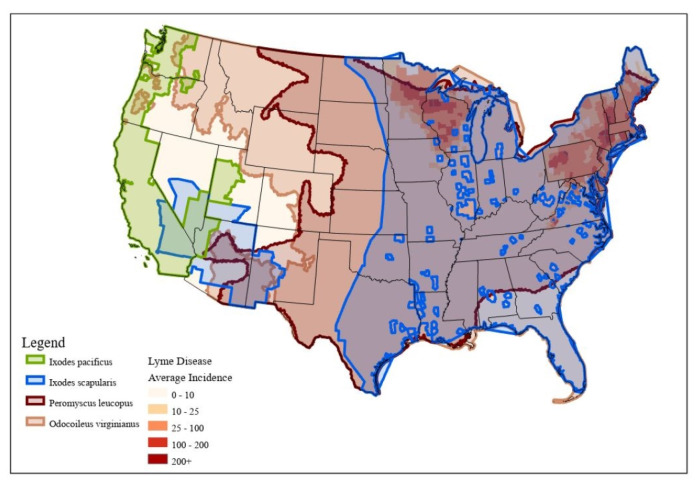
Species range and Lyme disease incidence rates in the U.S. *Ixodes pacificus*: western blacklegged tick; *Ixodes scapularis*: blacklegged tick; *Peromyscus leucopus*: white-footed deer mouse; *Odocoileus virginianus*: white-tailed deer.

We first conducted a nationwide analysis in order to attain a greater depth of understanding of how these associations can be particularly useful for targeting at-risk populations in the context of the expansion of the geographic ranges of vectors (Figure 2). Vectors included *I. scapularis* and *I. pacificus*, because these two species are responsible for spreading the bacteria into the human population, *P. leucopus*, which is one of the main reservoirs of *B. burgdorferi*, and *O. virginianus*, because this species along with other medium–large-sized mammals aids in tick survival [30]. We mapped the locations of high-risk LifeModes nationwide.

**Figure 3:**
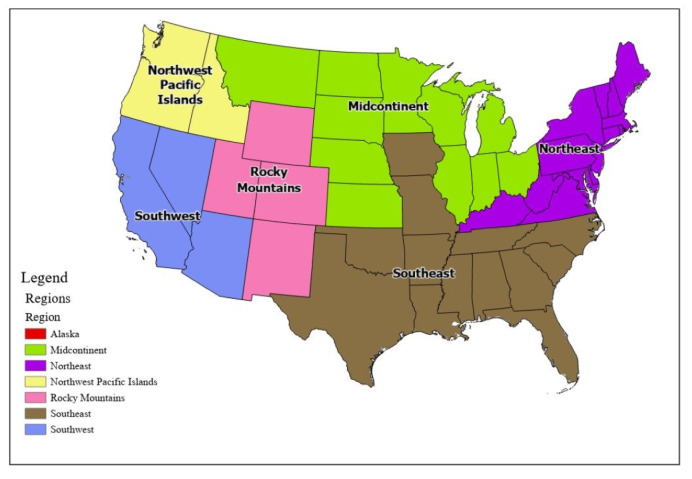
U.S. regions adapted from the USGS regional map. Northeast: CT, DE, KY, ME, MA, MD, NH, NJ, NY, PA, RI, VA, VT, and WV; Southeast: AL, AR, FL, GA, IA, LA, MS, MO, NC, OK, SC, TN, and TX; Midcontinent: IL, IN, KS, MN, MI, MT, NE, ND, OH, SD, and WI; Rocky Mountains: CO, NM, UT, and WY; Southwest: AZ, CA, and NV; Northwest and Pacific: HI, ID, OR, and WA; Alaska: AK.

Due to the extreme regional nature of Lyme disease incidence, we carried out our national-level analysis at the regional level. We divided the U.S. into seven regions that were adapted from the USGS regional map (https://www.usgs.gov/media/images/usgs-regional-map) (accessed on 4 August 2017). Figure 3 shows these regions by state boundaries.

#### Statistical Analysis

We used analysis of variance (ANOVA) [31] to determine whether there was any association between Lyme disease incidence and LifeModes. Our research question was “Is there a difference in average incidence rate among different LifeModes?” We further used analysis of means (ANOM) [32] and post hoc tests to determine which particular LifeModes had higher risk. Our research question was “Which LifeModes have incidence rates which are higher/lower than average?”.

Since there are many similarities and overlaps between lifestyle segments within the same LifeModes, and testing at the segment level would also drastically reduce sample sizes, curtailing the power of the statistical tests, we chose to use the broader Tapestry LifeModes, rather than lifestyle segments, for the statistical analysis. We ran Tukey’s HSD post hoc test with the following research questions: Which pairs of LifeModes have significantly different incidence rates? Which LifeModes could potentially have the maximum incidence rate?

The same statistical analyses from the national analysis (i.e., ANOVA, ANOM, and Tukey’s post hoc test) were carried out separately for each of these seven regions. We ran the ANOM test for the five regions where a significant difference was detected. Tukey’s HSD post hoc test could not be performed at the regional level, because some of the LifeModes had fewer than two counties within some of the regions. We mapped Lyme disease incidence with associated high-risk LifeModes in the Northeast, Southeast, Midcontinent, Rocky Mountain, and Southwest regions.

**Figure 4:**
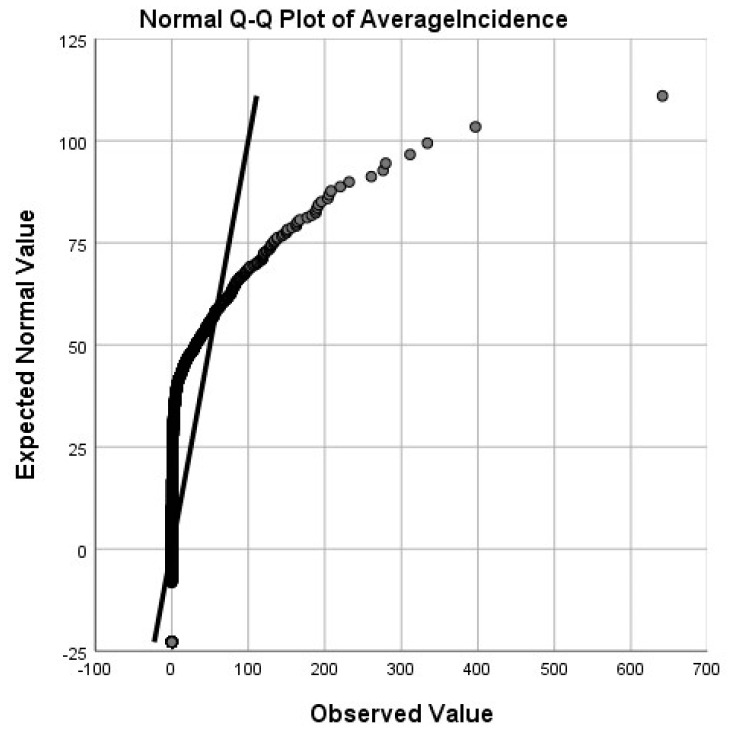
The quantile plot for the untransformed rates.

**Figure 5:**
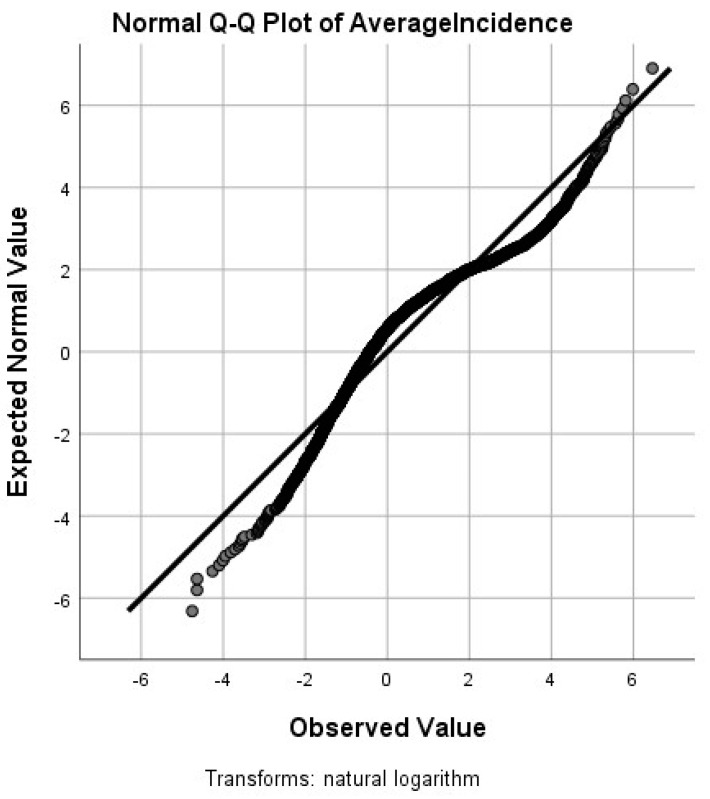
The quantile plot for the log-transformed rates.

Our national analysis included all of the counties in the United States. First, exploratory data analysis was performed to determine whether the Lyme disease incidence rates were normally distributed. Figure 4 shows the quantile plot for the untransformed rates, clearly indicating the severe right-skewness in the data; we used a log-transform to remedy this. Figure 5 shows the quantile plot for the log-transformed rates. As the Figure 5 plot is much closer to the normal distribution than the non-transformed data, we used the log-transformed data.

One-way ANOVA was performed with LifeModes as the factor variable and log-transformed incidence rates as the response variable to determine whether there were differences in average incidence rates between different LifeModes; Table 1 displays these results. The one-way ANOVA compares the means between the LifeModes and determines whether any of those means are statistically significantly different from one another. Specifically, it tests the null hypothesis:

“All means are equal”:

$$H_{o}:\;\mu_{1}=\mu_{2}=\mu_{3}=\cdots =\mu_{k}$$

where *µ* is the group mean and *k* is the number of groups. If the one-way ANOVA returns a statistically significant result, we accept the alternative hypothesis (H_A_) “Not all means are equal”, which is that there are at least two group means that are statistically significantly different from one another.

**Figure 6:**
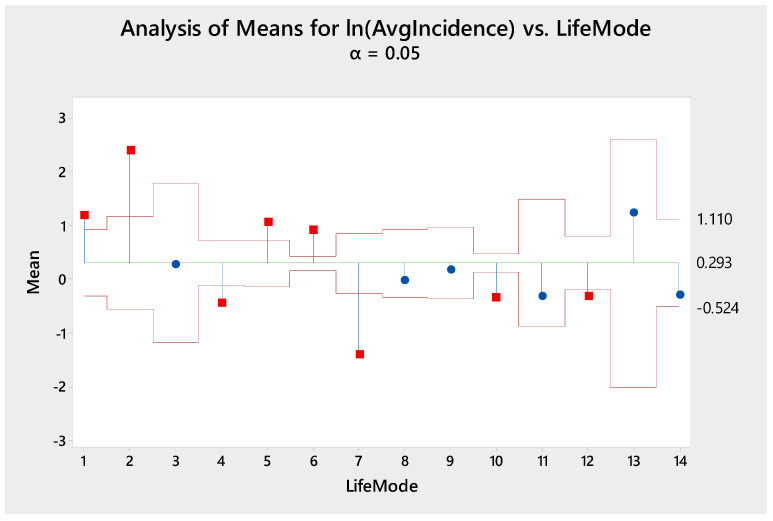
Analysis of means for average Lyme disease incidence vs. LifeModes.

Analysis of means (ANOM) was also performed to determine which LifeModes have incidence rates that are significantly above/below the overall mean incidence rate. The above table contains 95% confidence intervals for the mean incidence of each LifeMode. For each LifeMode with a confidence interval entirely above the overall mean (8.03 per 100,000), we can conclude that this LifeMode had an above average risk of Lyme disease. Similarly, for each LifeMode with a confidence interval entirely below the overall mean, we can conclude that this LifeMode had a below average risk of Lyme disease. Figure 6 displays a graphical representation of the analysis of means for Lyme disease incidence vs. LifeModes.

The one-way ANOVA cannot tell us which specific LifeModes were statistically significantly different from one another—it could only tell us that at least two groups were. Tukey’s HSD post hoc test determined which pairs of LifeModes were significantly different. We could also determine which LifeMode(s) had the highest incidence rate(s).

## 4. Results

### 4.1. National Analysis Results

The results of the one-way ANOVA analysis for Lyme disease incidence (ANOVA, *F*-value = 30.659, *p* < 0.001; Table 2) show a significant association between Lyme disease incidence and LifeModes at the national level for an 18-year period from 2000 to 2017.

We then used analysis of means (ANOM) to investigate which LifeModes had higher risk. As Table 3 states, LifeModes 1 (Affluent Estates), 2 (Upscale Avenues), 5 (GenXurban), and 6 (Cozy Country Living) exhibited a significantly higher mean incidence rate than the overall mean. LifeModes 4 (Family Landscape)*,* 7 (Ethnic Enclaves)*,* 10 (Rustic Outposts)*,* and 12 (Hometown) had a significantly lower mean incidence rate than the overall mean. Appendix B shows an in-depth description of the lifestyle traits of the high- and low-risk LifeModes and lifestyle segments influencing Lyme disease morbidity.

The results of Tukey’s HSD test indicated that either LifeMode 1 (Affluent Estates) or LifeMode 2 (Upscale Avenues) had the highest Lyme disease incidence rate.

Our systematic review of the households that fall within these high- and low-risk LifeModes revealed commonalities of lifestyle preferences and life stages that could affect the risk of contracting Lyme disease. Appendix C shows a county-based summary of LifeModes associated with high incidence and low incidence, along with their predominant lifestyle traits. Single-family home ownership, living in old suburbs and/or urban settings with older homes, being active in sports and outdoor recreation, and engaging in outdoor activities such as gardening and maintaining lawns are common lifestyle preferences among both high- and low-risk households. This explains the risk of tick bites in Lyme-disease-endemic areas. What makes the high-risk households different to the low-risk households is that they are generally older and wealthier individuals, being predominantly white, from high-income neighborhoods, college-educated professionals, enthusiastic travelers, and active in outdoor recreational sports such as walking, jogging, hiking, etc. Low-risk households have a wide range of ages, with less income, and varying net worth depending on how well they budget. These individuals are ethnically diverse (e.g., Hispanic families), from low-income neighborhoods, educated to high-school level or less, and engage in outdoor activities such as hunting, fishing, lawn maintenance, and vegetable gardening; however, they also partake in many indoor activities. Low-risk households are not as enthusiastic travelers as high-risk households, but they take trips to theme parks, water parks, or the zoo.

### 4.2. Climate and Habitat Variables of High-Risk LifeModes

**Figure 7:**
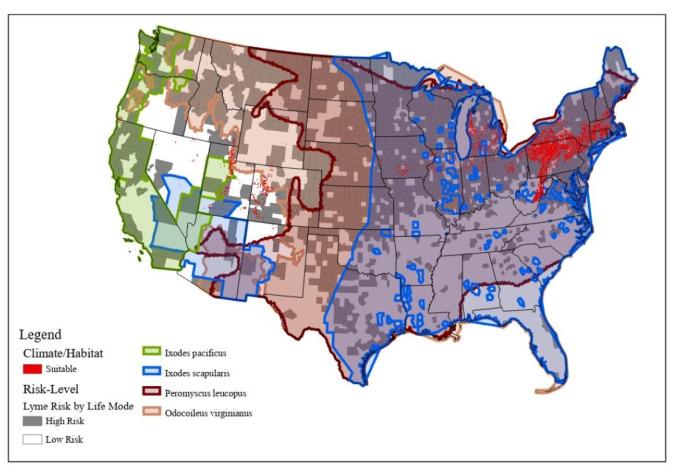
At-risk LifeModes and climate and habitat suitability with species range. *Ixodes pacificus*: western blacklegged tick; *Ixodes scapularis*: blacklegged tick; *Peromyscus leucopus*: white-footed deer mouse; *Odocoileus virginianus*: white-tailed deer.

Figure 7 shows the high-risk life modes and suitable climate and habitat variables overlain with species ranges for western blacklegged ticks, blacklegged ticks, white-tailed deer, and white-footed deer mice. High-risk LifeModes are heavily concentrated in the Mid-Atlantic area (i.e., New York, Connecticut, and Pennsylvania), and one in the upper Midwest (i.e., Minnesota and Wisconsin). High-risk-LifeMode areas are associated with the presence of white-footed deer mice (which transmit bacteria to ticks) and white-tailed deer (which are associated with tick life cycles). These are Lyme-disease-endemic areas with environmental conditions conducive to tick habitats and an abundance of hosts for the ticks. We examined climatic and habitat variables that influenced the distribution of LD in high-risk-LifeMode areas such as heavily forested areas, overwintering areas, and areas with high amounts of annual precipitation (e.g., snow residence). We combined these three layers into one suitability layer. Food is essential to survival, because ticks feed on other species to survive. Areas that have food available for their hosts (e.g., *Peromyscus leucopus*, *Odocoileus Virginianus*, and medium-sized mammals) are important to note, including forested areas—especially oak forests, because they provide much of this food [30]. We also extracted overwintering areas whose average minimum temperatures remain higher than −10 degrees Celsius during the winter months (December–February), and with a snow residence time of more than 50 days [33]. These are important climate and habitat factors that affect ticks’ development, survival, and host-seeking behavior, as well as strongly influencing tick abundance. The proportion of ticks infected with the Lyme disease spirochete, *Borrelia burgdorferi*, depends on the abundance of hosts for the ticks and the capacity of tick hosts to serve as *B. burgdorferi* reservoirs.

Counties that consist predominantly of households engaged in at-risk LifeModes are shown in gray. These maps reveal that a significant portion of the United States consists of at-risk LifeModes. The Lyme-disease-endemic areas (orange dots) are within areas that have the heaviest concentrations of at-risk LifeModes.

The high-risk LifeModes of Affluent Estates, Upscale Avenues, GenXurban, and Cozy Country Living had high percentages of suitable area coverage conducive to tick survival. We also found that there were high-risk traveler households that did not live in endemic areas, but contracted the disease elsewhere (Figure 7). Cases are reported according to county of residence, not by county of exposure. For example, major concentrations of high-risk Urban Chic households are found in urban areas on the Northern and Southern California coasts, as well as along the east coast; they travel extensively, visit national parks, stay active; and for fitness they engage in downhill skiing, backpacking, hiking, biking, yoga, aerobics, tennis, and weightlifting.

**Figure 8:**
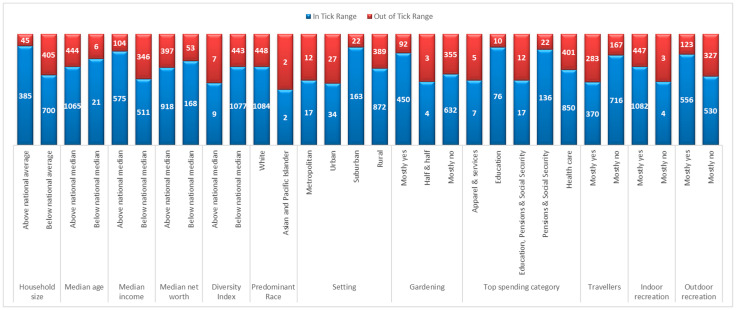
Demographic and behavioral traits for counties with high-risk LifeModes. Blue indicates the number of counties located within *Ixodes scapularis* (blacklegged tick), *Ixodes pacificus* (western blacklegged tick), or any overlap of the two ranges. Red indicates the number of counties located outside of their ranges.

We further conducted trait analysis of the high-risk LifeModes to see which traits were related to higher Lyme disease incidence. Figure 8 shows demographic and behavioral traits for high-risk LifeModes determined from our analyses. It can be seen that counties of high-risk LifeModes are within the ticks’ range for each trait category. Most counties at high risk of Lyme disease have household sizes below the national average, with a median net worth generally higher than average. The diversity in these areas is generally lower, with the predominant race being White or Asian and Pacific Islanders. These individuals are also generally older, live in suburban or rural settings, and spend most of their budget on healthcare. Within the ranges of either of the ticks, individuals are not well traveled. However, when looking at individuals at high risk of Lyme disease outside of tick ranges, one finds an increase in travelers. These individuals also enjoy both indoor and outdoor recreation, but there were far fewer individuals who were interested in outdoor recreation outside of tick ranges than within tick ranges.

One-way ANOVA and Tukey’s HSD post hoc test for Lyme disease incidence by demographic and social traits were conducted in order to determine which traits were related to higher Lyme disease incidence. Results from the ANOVA tests can be seen in Table 4. Lifestyle traits whose mean incidence rates significantly differed across groups included area setting, household size, median age, median income, median net worth, predominant career field, predominant spending category, gardening, travelling, interest in indoor recreational activities, and interest in outdoor recreational activities.

Table 5 shows significant pairwise comparisons between lifestyle traits in order to determine which traits differ the most. Individuals who live in suburban areas are at higher risk of Lyme disease than those in semi-rural or urban areas. Suburban areas tend to have bigger backyards, which could lead to more outdoor activities, such as lawn maintenance, gardening, etc. Individuals may not prepare for tick encounters when conducting shorter trips in their backyards, which could lead to increased tick exposure. Households in high-risk counties often also have more people living in them, increasing the amount of people with similar behaviors in these bigger yards. Furthermore, individuals at higher risk are older, with higher income and net worth. When looking more closely at the predominant career fields, there are many instances where people who work in management, along with a combination of other career fields, have higher mean incidence rates for Lyme disease. People who spend a majority of their expenses on healthcare have lower incidence rates than those who spend a majority of their expenses on education. Areas that have individuals who garden along with individuals who do not garden tend to have higher mean Lyme disease incidence rates than those who mostly do not garden or those who mostly do garden. If it is not a social norm to garden, people may not understand what kind of precautions are needed in order to reduce exposure to tick bites while gardening. Furthermore, people who travel are at higher risk of Lyme disease than those who do not. This makes sense, because some people may not have ticks in their region and, thus, fail to take proper precautions; when travelling, they are unaware of the tick prevention measures necessary in their destination. Finally, people who are interested in indoor recreation have higher mean incidence rates than those who are not. People who are interested in outdoor recreation are well educated in the proper prevention techniques needed when they are outside, while people who mainly enjoy indoor recreation may not know or practice these techniques.

### 4.3. Regional Analysis Results

We found that there was a difference in the average Lyme disease incidence among the different LifeModes in the Northeast, Southeast, Midcontinent, Rocky Mountain, and Southwest regions, as shown in Table 6. The United States has two major Lyme disease hotspots: one in the Mid-Atlantic area (i.e., New York, Connecticut, and Pennsylvania), and one in the upper Midwest (i.e., Minnesota and Wisconsin). The risk of Lyme disease is very low throughout the rest of the United States.

**Figure 9:**
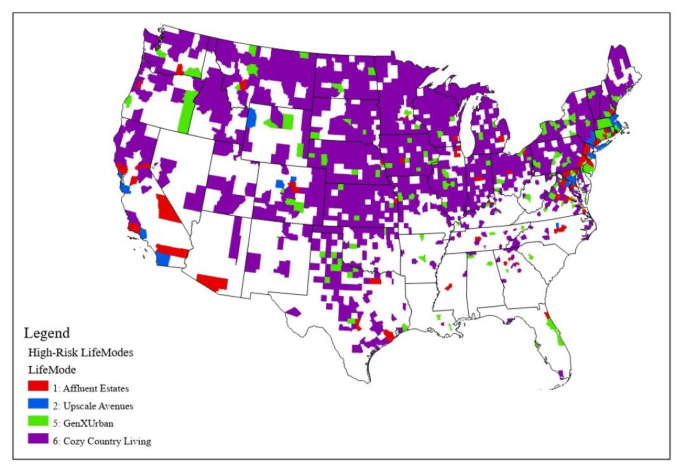
High-risk LifeModes with regional boundaries.

We also found that the ANOM test results reflected the location-dependent nature of the relationship between lifestyle and Lyme disease (Table 7). For example, LifeMode 1 (Affluent Estates) experiences above average Lyme disease incidence in the Northeast but below average incidence in the Rocky Mountains region. LifeMode 2 (Upscale Avenues) experiences above average incidence in the Northeast and Rocky Mountains. LifeMode 4 (Family Landscape) experiences below average incidence in the Southeast. LifeMode 6 (Cozy Country Living) experiences above average incidence in the Northeast, Southeast, Midcontinent, and Southwest. LifeMode 7 (Ethnic Enclaves) experiences below average incidence in the Southeast and Southwest. LifeMode 10 (Rustic Outposts) experiences below average incidence in the Northeast. LifeMode 11 (Midtown Singles) experiences below average incidence in the Rocky Mountains. LifeMode 12 (Hometown) experiences below average incidence in the Southeast and Midcontinent regions. Figure 9 shows high-risk LifeModes in each region.

It is interesting to note that while LifeModes 1 (Affluent Estates), 2 (Upscale Avenues), 5 (GenXurban), and 6 (Cozy Country Living) were all found to be associated with higher Lyme disease incidence nationwide, LifeMode 5 (GenXurban) was not significantly higher in any of the individual regions, LifeModes 1 (Affluent Estates) was significantly higher only in the Northeast, and LifeMode 2 (Upscale Avenues) was significantly higher only in the Northeast and Rocky Mountains. It is likely that the high incidence rates in the Northeast skewed the nationwide statistics. It is important to note that LifeMode 6 (Cozy Country Living) is consistently associated with higher incidence rates across most of the nation. It is recommended that local policy decisions outside of the Northeast should be based on our regional results for the location in question rather than on our national results.

## 5. Discussion

If we are to limit the impact of emerging Lyme disease on human health in the U.S., the appropriate prevention measures should be implemented and targeted towards the at-risk populations in the high-risk locations [9]. Prevention measures include personal protection, environmental management for tick control, and community-based interventions such as rodent-targeted vaccines (RTVs), as many small rodents are carriers of *B. Burgdorferi* [9], e.g., *P. leucopus* [5], *Sciurus griseus* [34], *Zapus hudsonius*, and *Ictidomys tridecemlineatus* [35], among others. The common approach for defining human populations at risk of Lyme disease has been identifying endemic locations and predicting the occurrence of vectors using risk maps. In many cases, potential geographic distributions of vectors have been predicted using statistical associations between climate or landscape variables (or their remote-sensed proxies), which are likely to be associated with vector survival and/or reproduction and, thus, the observed occurrence of vectors [36,37]. The human populations at risk are, at least, defined in part by the geographic occurrence of the arthropod vectors, whose existence is tightly linked to climatic variables on a continental scale [38,39], as well as to suitable habitats on a more local geographic scale [36].

Even though environmental and climatic factors are driving the increase in the Lyme disease vectors in the emerging geographic areas, human social behaviors—such as lifestyle preferences—also affect the risk of contracting Lyme disease. Our findings contributed a human behavioral aspect to these investigations, and led to geographically identified at-risk target populations based on lifestyle preferences [1]. Our resultant risk maps show at-risk LifeMode households in localized endemic areas, and are potentially very useful to guide public health policy and target surveillance and intervention activities [1]. We can predict which households will be at risk of Lyme disease in new emerging endemic areas based on our findings on lifestyle preferences.

With a greater depth of understanding of these at-risk households based on lifestyle, we can further explore the localized households in the risk maps that were the result of predicting expansion of the geographic ranges of vectors. For example, household-level findings provide prevention opportunities for localized interventions such as the deployment of rodent-targeted vaccines (RTVs). RTVs have been successful in preventing *B. burgdorferi* infection in rodent reservoirs and host-seeking ticks by disrupting transmission cycles [40,41,42]. At-risk households are potential grounds to deploy RTVs that can block or significantly reduce the chance/ability of arthropod vectors to become infected with and transmit disease-causing pathogens to uninfected reservoirs or humans [40,41,42,43,44].

Protection and prevention products such as tick-repellent products could be more efficiently marketed to these at-risk populations/households based on their lifestyle preferences. For example, Affluent Estates and Upscale Avenues households are early adapters of new products and technology; they enjoy the outdoors, and are health conscious; they have high rates of homeownership that would make them likely to invest in chemical control products. This lifestyle segmentation not only provides information on how to market to these at-risk households, but can also be used to conduct more efficient health intervention, prevention, and treatment. For example, public health messages and clinical information could be issued to the public and medical practitioners in these at-risk households for better assistance in clinical diagnoses. Lifestyle segmentation can provide clues for physicians as to how to more properly diagnose patients, in much the same way as these data enable more efficient marketing to consumers.

Clinical trials of new vaccines for Lyme disease can recruit patients from high-risk households based on their lifestyle preferences, and can determine their overall motivation to engage in clinical research. High-risk LifeModes and their locations are clearly the areas in the U.S. where the public might benefit from a Lyme disease vaccine. Clinical trials and digital advertising campaigns can use these at-risk households as georeferencing targets and tailor their recruitment campaigns and marketing efforts based on these households’ lifestyle preferences. These areas are also targeted areas to increase awareness of a vaccine among the public and clinicians in order to prevent Lyme disease in the United States.

We also found that there is a location-dependent relationship between lifestyle and infection; that is, there were counties that had LifeModes and segmentations that were associated with high Lyme disease incidence, but no actual incidence was recorded in those counties. Therefore, at-risk-LifeMode households might not constitute a risk in non-endemic areas when climate and habitat conditions are not suitable for vectors. Some counties that have high Lyme disease incidence in non-endemic areas include at-risk lifestyle populations/households such as Urban Chic, because these are enthusiastic travelers who might have visited endemic areas and contracted the disease during their leisurely outdoor activities, such as hunting and hiking. Many hunting activities occur in forested areas for wild game associated with *B. burgdorferi* transmission, which creates a relevant risk of exposure for hunters [45]. Educational tools, clinical trials campaigns, and public health messages—such as vaccine awareness—could be issued to these at-risk lifestyle populations/households in those non-endemic areas in order to make prevention—such as personal protection—part of their planning before visiting endemic areas for camping, hiking, hunting, and other outdoor activities.

## 6. Conclusions

We conclude that there needs to be more research done on translating science into real-world solutions. Given that virtually every household in the U.S. has been assigned a lifestyle segment, linking segments to geographically identified patients (e.g., incidence, morbidity) in healthcare delivery systems could support the ability to estimate morbidity levels for various conditions and, subsequently, predict the demand for health services. Establishing this link can also allow for more efficient, more targeted, and cost-effective health care [1]. Our methodology for Lyme disease in this paper, and for COVID-19 in a previous methodological paper [1], aims to be a prototype for converting information on lifestyles into the incidence and prevalence of health conditions, along with the demand for health services.

## Figures and Tables

**Table 1:** Analysis of variance.

Source	DF	Adj SS	Adj MS	F-Value	*p*-Value
LifeMode	13	144,073	11,082.5	13.79	0.000
Error	3121	2,508,283	803.7		
Total	3134	2,652,356			

Pooled standard deviation = 28.3492.

**Table 2:** Results of the one-way ANOVA for Lyme disease clusters and incidence.

ANOVA	F-Value	*p*-Value ***	Significant?
Lyme disease incidence	30.659	<0.001	Yes

*** *p* < 0.001.

**Table 3:** The results of the ANOM tests.

Code	LifeMode	Risk Level	Lyme Disease Cases per 100,000	% Suitable (Climate and Habitat)
N/A	Nationwide Average	N/A	8.03	1.9
1	Affluent Estates	High	30.14	5.4
2	Upscale Avenues	High	36.10	4.2
3	Uptown Individuals	Low	3.87	2.8
4	Family Landscapes	Low	3.95	0.9
5	GenXurban	High	13.78	6.0
6	Cozy Country Living	High	12.15	2.5
7	Ethnic Enclaves	Low	0.44	0.2
8	Middle Ground	Low	3.94	1.2
9	Senior Styles	Low	5.23	0.1
10	Rustic Outposts	Low	2.14	0.8
11	Midtown Singles	Low	5.28	0.5
12	Hometown	Low	2.54	0.5
13	Next Wave	Low	12.92	1.6
14	Scholars and Patriots	Low	2.11	3.1

**Table 4:** Results of the one-way ANOVA tests for Lyme disease incidence by lifestyle trait.

ANOVA	F-Value	*p*-Value ***	Significant?
Setting	3.366	0.005	Yes
Married couples	1.853	0.158	No
Children	1.104	0.332	No
Household size	3.888	0.049	Yes
Median age	6.687	0.010	Yes
Median income	18.19	<0.001	Yes
Median net worth	64.67	<0.001	Yes
Diversity index	2.725	0.099	No
Predominant race	1.301	0.247	No
Predominant career field	12.65	<0.001	Yes
Predominant spending category	3.966	<0.001	Yes
DIY home improvement	3.308	0.069	No
Gardeners	5.029	0.007	Yes
Travelers	19.44	<0.001	Yes
Indoor recreation	5.18	0.023	Yes
Outdoor recreation	2.116	0.146	No

*** *p* < 0.001.

**Table 5:** Pairwise comparisons for Lyme disease incidence by lifestyle trait.

Demographic Trait	Significant Comparison	Mean Difference	*p*-Value
Setting	Suburban vs. Semi-rural	32.8	0.039
	Urban vs. Suburban	−33.0	0.026
Household size	Above national median vs. Below national median	9.4	0.049
Median age	Above national median vs. Below national median	18.7	0.010
Median income	Above national median vs. Below national median	22.7	<0.001
Median net worth	Above national median vs. Below national median	35.2	<0.001
Predominant career field	Office and administrative support and food preparation and serving vs. Office and administrative support only	57.4	0.006
	Management, office and administrative support, and sales vs. Construction and extraction, and office and administrative support	73.7	<0.001
	Production and office and administrative support vs. Management	−58.0	0.011
	Management, office and administrative support, and sales vs. Management and office and administrative support	59.6	0.001
	Office and administrative support vs. Management, office and administrative support, and sales	−51.1	<0.001
	Production and office and administrative support vs. Management, office and administrative support, and sales	−74.3	<0.001
Top spending category	Health care vs. Education	−24.9	0.002
Gardeners	No vs. Mixed	−80.4	0.009
	Yes vs. Mixed	−74.3	0.017
Travelers	Yes vs. No	43.5	<0.001
Interested in indoor recreation	Yes vs. No	18.9	0.023

**Table 6:** Results of the one-way ANOVA test for Lyme disease incidence by region.

Region	F-Value	*p*-Value	Significant?
Northeast	13	<0.001	Yes
Southeast	12.42	<0.001	Yes
Midcontinent	2.695	0.002	Yes
Rocky Mountains	7.305	<0.001	Yes
Southwest	3.175	0.001	Yes
Northwest and Pacific	1.197	0.307	No
Alaska	0.986	0.488	No

**Table 7:** Results of the ANOM test by region.

Region	Mean Lyme Disease Incidence	High-Risk LifeModes	Low-Risk LifeModes
Northeast	29.052	1 Affluent Estates,	10 Rustic Outposts
		2 Upscale Avenues,	
		6 Cozy Country Living	
Southeast	0.6918	6 Cozy Country Living	4 Family Landscape
			7 Ethnic Enclaves
			12 Hometown
Midcontinent	8.9868	6 Cozy Country Living	12 Hometown
Rocky Mountains	0.1235	2 Upscale Avenues,	1 Affluent Estates,
			11 Midtown Singles
Southwest	0.7373	6 Cozy Country Living	7 Ethnic Enclaves

## Data Availability

Raw data “Lyme disease rates” were derived from the CDC Wonder database available in the public domain at https://www.cdc.gov/lyme/stats/survfaq.html, accessed on 23 November 2021. The authors confirm that the data supporting the findings of this study are available within the article. The GIS data and maps of High and Low Risk LifeModes are not publicly available due to commercialization of research findings.

## Appendix A

**Table A1:** Dominant traits for each LifeMode adapted from ESRI Tapestry 2020 Summary Table (http://downloads.esri.com/esri_content_doc/dbl/us/2020TapestryLifeModeGroupSummaryTables.pdf, accessed on 5 January 2020).

Demographics										
**LifeMode Name**	**Code**	**Counties**	**Households**	**Population**	**Household Type**	**Average Household Size**	**Diversity Index**	**Median Age**	**Median Income**	**Median Net Worth**
Affluent Estates	1	71	12,589,391	36,589,686	Married couples	2.88	46.2	43.1	USD 129,800	USD 715,900
Upscale Avenues	2	41	7,030,246	19,167,649	Married couples	2.69	67.7	40.9	USD 105,000	USD 268,400
Uptown Individuals	3	13	4,848,096	9,282,562	Singles	1.85	66.2	35.3	USD 89,700	USD 44,500
Family Landscape	4	159	9,571,331	27,460,541	Married couples	2.85	55.9	37.2	USD 81,100	USD 184,000
GenXurban	5	163	14,252,029	34,994,853	Married couples	2.41	43.3	43.9	USD 66,800	USD 157,800
Cozy Country Living	6	1261	15,175,430	38,619,224	Married couples	2.51	28.1	45.5	USD 62,700	USD 163,200
Ethnic Enclaves	7	106	9,019,686	30,363,455	Married couples	3.34	82.8	32.1	USD 59,500	USD 79,800
Middle Ground	8	80	13,638,949	33,367,170	Mixed	2.40	70.4	36.5	USD 54,500	USD 36,800
Senior Styles	9	69	7,315,711	14,828,033	Mixed	1.94	49.1	58.3	USD 50,800	USD 111,600
Rustic Outposts	10	965	10,431,913	27,770,247	Married couples	2.59	50.5	41.0	USD 46,300	USD 75,400
Midtown Singles	11	21	7,755,759	18,806,661	Singles	2.37	79.2	31.3	USD 39,100	USD 12,900
Hometown	12	141	7,628,789	19,341,859	Mixed	2.47	66.3	38.4	USD 37,300	USD 23,000
Next Wave	13	5	4,795,987	16,045,217	Mixed	3.30	89.6	30.0	USD 39,900	USD 13,300
Scholars and Patriots	14	45	2,028,867	6,609,014	Mixed	2.27	60.0	22.9	USD 33,300	USD 10,900
**Education**										
**LifeMode Name**	**Code**	**No High-School Diploma**		**High-School Diploma/GED**		**Some College**		**Associate Degree**	**Bachelor’s Degree**	**Graduate Degree**
Affluent Estates	1	3.2%		13.4%		15.6%		7.5%	33.5%	26.9%
Upscale Avenues	2	6.3%		18.4%		17.0%		8.0%	29.0%	21.3%
Uptown Individuals	3	4.9%		9.4%		11.4%		4.6%	37.8%	31.9%
Family Landscape	4	7.2%		25.1%		22.8%		10.6%	22.4%	11.7%
GenXurban	5	7.0%		27.2%		21.4%		10.0%	21.3%	13.1%
Cozy Country Living	6	8.7%		33.7%		21.8%		10.5%	16.4%	8.9%
Ethnic Enclaves	7	21.8%		27.9%		20.9%		8.3%	14.6%	6.4%
Middle Ground	8	11.5%		26.1%		20.9%		8.7%	20.6%	12.1%
Senior Styles	9	9.4%		26.2%		20.6%		7.9%	20.8%	15.2%
Rustic Outposts	10	16.9%		38.6%		20.9%		8.6%	9.9%	5.1%
Midtown Singles	11	14.6%		28.6%		22.6%		8.5%	16.9%	8.7%
Hometown	12	15.9%		36.7%		23.0%		8.5%	10.6%	5.3%
Next Wave	13	32.6%		28.9%		16.7%		5.8%	11.3%	4.7%
Scholars and Patriots	14	6.2%		16.9%		21.2%		8.2%	26.4%	21.1%

## Appendix B

**Table A2:** In-depth description of the lifestyle traits of the high- and low-risk LifeModes and lifestyle segments influencing Lyme disease morbidity, adapted from ESRI Tapestry 2020 Summary Table (http://downloads.esri.com/esri_content_doc/dbl/us/2020TapestryLifeModeGroupSummaryTables.pdf, accessed on 5 January 2020).

High-Risk									
**LifeMode Name**	**Segment Name**	**Households**	**Population**	**Household Type**	**Average Household Size**	**Diversity Index**	**Median Age**	**Median Income**	**Median Net Worth**
Affluent Estates	1A—Top Tier	2,111,573	6,050,994	Married couples	2.83	39.7	47.9	USD 185,300	USD 1,408,800
	1B—Professional Pride	2,055,809	6,433,030	Married couples	3.12	46.7	40.7	USD 151,700	USD 888,100
	1C—Boomburbs	2,232,537	7,257,017	Married couples	3.24	65.0	34.1	USD 123,900	USD 383,300
	1D—Savvy Suburbanites	3,746,675	10,678,017	Married couples	2.83	38.3	45.6	USD 116,900	USD 636,300
	1E—Ex Urbanites	2,442,797	6,170,628	Married couples	2.48	37.0	51.6	USD 110,300	USD 632,300
Upscale Avenues	2A—Urban Chic	1,639,592	3,999,202	Married couples	2.39	49.9	43.6	USD 120,600	USD 352,900
	2B—Pleasantville	2,709,951	7,865,434	Married couples	2.87	62.5	43	USD 103,500	USD 375,800
	2C—Pacific Heights	872,917	2,789,950	Married couples	3.16	74.4	43.1	USD 104,300	USD 302,400
	2D—Enterprising Professionals	1,807,786	4,513,063	Married couples	2.48	74.1	35.7	USD 97,300	USD 110,100
GenXurban	5A—Comfortable Empty Nesters	3,087,193	7,809,376	Married couples	2.50	35.0	48.6	USD 80,300	USD 295,500
	5B—In Style	2,828,681	6,739,676	Married couples with no kids	2.34	41.9	42.4	USD 79,800	USD 162,100
	5C—Parks and Rec	2,475,722	6,245,809	Married couples	2.50	53.0	41.4	USD 66,600	USD 124,000
	5D—Rustbelt Traditions	2,748,758	6,817,742	Married couples	2.46	49.0	39.5	USD 55,800	USD 97,500
	5E—Midlife Constraints	3,111,675	7,382,250	Married couples with no kids	2.29	37.9	47.3	USD 57,300	USD 135,400
Cozy Country Living	6A—Green Acres	4,086,329	11,064,683	Married couples	2.69	27.9	44.5	USD 83,900	USD 272,500
	6B—Salt of the Earth	3,611,849	9,375,498	Married couples	2.57	21.1	44.6	USD 61,600	USD 164,100
	6C—The Great Outdoors	1,985,000	4,905,828	Married couples	2.43	37.0	47.9	USD 62,100	USD 155,600
	6D—Prairie Living	1,339,996	3,407,393	Married couples	2.50	25.8	44.6	USD 60,300	USD 155,600
	6E—Rural Resort Dwellers	1,280,816	2,873,228	Married couples with no kids	2.21	24.6	54.9	USD 55,600	USD 163,900
	6F—Heartland Communities	2,871,438	6,992,594	Married couples	2.39	33.3	42.5	USD 46,700	USD 71,500
**Low-Risk**									
**LifeMode Name**	**Segment Name**	**Households**	**Population**	**Household Type**	**Average Household Size**	**Diversity Index**	**Median Age**	**Median Income**	**Median Net Worth**
Family Landscape	4A—Soccer Moms	3,719,727	11,053,960	Married couples	2.96	52.9	37.1	USD 100,500	USD 284,700
	4B—Home Improvement	2,145,166	6,166,197	Married couples	2.86	67.5	38.2	USD 78,200	USD 181,300
	4C—Middleburg	3,706,438	10,240,384	Married couples	2.74	50.6	36.6	USD 66,900	USD 119,000
Ethnic Enclaves	7A—Up and Coming Families	3,211,195	10,051,661	Married couples	3.11	75.1	31.8	USD 80,000	USD 131,500
	7B—Urban Villages	1,311,784	5,002,060	Married couples	3.78	86.2	34.4	USD 71,600	USD 124,400
	7C—American Dreamers	1,857,195	5,962,189	Married couples	3.19	84.7	32.9	USD 55,200	USD 64,200
	7D—Barrios Urbanos	1,309,286	4,789,156	Married couples	3.62	80.8	29.2	USD 43,200	USD 31,300
	7E—Valley Growers	304,463	1,232,632	Married couples	3.96	84.7	27.7	USD 38,300	USD 16,300
	7F—Southwestern Families	1,025,763	3,325,757	Married couples	3.19	64.7	34.8	USD 34,300	USD 19,500
Rustic Outposts	10A—Southern Satellites	3,988,291	10,719,631	Married couples	2.66	42.0	40.7	USD 52,900	USD 99,100
	10B—Rooted Rural	2,488,566	6,283,674	Married couples	2.47	30.3	45.7	USD 46,700	USD 96,000
	10C—Diners and Miners	821,345	2,142,316	Married couples	2.53	44.0	41.8	USD 44,500	USD 68,600
	10D—Down the Road	1,457,886	4,080,295	Married couples	2.75	73.0	35.4	USD 41,900	USD 40,600
	10E—Rural Bypasses	1,675,825	4,544,331	Married couples	2.54	61.1	40.8	USD 35,900	USD 34,400
Hometown	12A—Family Foundations	1,292,784	3,536,499	Singles	2.70	43.7	40	USD 45,800	USD 58,200
	12B—Traditional Living	2,405,568	6,102,717	Married couples	2.50	57.6	36	USD 42,600	USD 33,800
	12C—Small Town Simplicity	2,314,916	5,451,181	Singles	2.25	52.6	41.1	USD 35,200	USD 18,900
	12D—Modest Income Homes	1,615,521	4,251,462	Singles	2.55	34.3	37.5	USD 26,700	USD 13,300

## Appendix C

**Table A3:** County-based summary of LifeModes associated with high incidence and low incidence, along with their predominant lifestyle traits.

Tapestry Segmentation for High Incidence of Lyme Disease									
**LifeMode Name**	**Dominant Segment**	**Number of Counties with High Incidence**	**Population**	**Setting**	**Predominant Race**	**Interest in Gardening**	**Interest in Travelling**	**Interest in Indoor Recreation**	**Interest in Outdoor Recreation**
Affluent Estates	1A—Top Tier	3	2,402,683	Suburban	White	No	Yes	Yes	No
	1B—Professional Pride	1	524,989	Suburban	White	No	Yes	Yes	No
	1C—Boomburbs	2	562,551	Suburban	White	No	No	Yes	No
	1D—Savvy Suburbanites	18	4,486,279	Suburban	White	No	Yes	Yes	No
	1E—Ex Urbanites	2	191,412	Suburban	White	No	Yes	Yes	No
Upscale Avenues	2A—Urban Chic	1	11,399	Suburban	White	About half and half	No	No	Yes
	2B—Pleasantville	14	10,233,995	Suburban	White	Yes	Yes	Yes	Yes
	2C—Pacific Heights	1	476,143	Urban	Asian and Pacific Islander	No	No	Yes	Yes
	2D—Enterprising Professionals	5	2,754,881	Suburban	White	Yes	Yes	Yes	Yes
GenXurban	5A—Comfortable Empty Nesters	4	1,534,893	Suburban	White	No	Yes	Yes	Yes
	5B—In Style	13	3,644,811	Metropolitan	White	No	No	Yes	No
	5C—Parks and Rec	21	7,661,155	Suburban	White	No	No	Yes	No
	5D—Rustbelt Traditions	3	385,848	Suburban	White	No	Yes	Yes	No
	5E—Midlife Constants	7	711,765	Urban	White	No	No	Yes	No
Cozy Country Living	6A—Green Acres	44	4,974,044	Rural	White	No	No	Yes	No
	6B—Salt of the Earth	53	3,644,805	Rural	White	Yes	No	Yes	Yes
	6C—The Great Outdoors	34	1,706,308	Rural	White	Yes	Yes	Yes	Yes
	6D—Prairie Living	22	437,400	Rural	White	No	Yes	Yes	No
	6E—Rural Resort Dwellers	36	833,376	Rural	White	Yes	No	Yes	No
	6F—Heartland Communities	31	1,585,535	Rural	White	No	No	Yes	Yes
**Tapestry Segmentation for Low Incidence of Lyme Disease**									
**LifeMode Name**	**Segment Name**	**Number of Counties with High Incidence**	**Population**	**Setting**	**Predominant Race**	**Interest in Gardening**	**Interest in Travelling**	**Interest in Indoor Recreation**	**Interest in Outdoor Recreation**
Family Landscape	4A—Soccer Moms	24	6,819,435	Suburban	White	No	No	Yes	No
	4B—Home Improvement	4	1,325,371	Suburban	White	No	No	Yes	Yes
	4C—Middleburg	96	11,650,487	Semi-rural	White	No	About half and half	Yes	No
Ethnic Enclaves	7A—Up and Coming Families	36	24,899,099	Suburban	White	No	No	No	No
	7B—Urban Villages	3	5,418,585	Urban	Hispanic	No	No	Yes	No
	7C—American Dreamers	5	5,566,295	Urban	White, Hispanic	No	No	Yes	No
	7D—Barrios Urbanos	10	4,217,326	Urban	Hispanic	No	No	Yes	No
	7E—Valley Growers	8	3,387,636	Urban	Hispanic	No	No	Yes	No
	7F—Southwestern Families	20	8,235,115	Urban, Suburban	Hispanic	Yes	No	Yes	Yes
Rustic Outposts	10A—Southern Satellites	210	14,222,472	Rural	White	No	No	Yes	Yes
	10B—Rooted Rural	178	4,485,653	Rural	White	Yes	No	Yes	Yes
	10C—Diners and Miners	62	1,504,617	Rural	White	Yes	No	No	Yes
	10D—Down the Road	14	1,171,061	Semi-rural	White	No	No	Yes	No
	10E—Rural Bypasses	103	2,885,518	Rural	White, Black	No	No	Yes	No
Hometown	12A—Family Foundations	4	2,175,757	Metropolitan	Black	Yes	No	Yes	Yes
	12B—Traditional Living	42	7,677,951	Urban	White	No	No	Yes	Yes
	12C—Small Town Simplicity	32	1,106,914	Semi-rural	White	No	No	Yes	No
	12D—Modest Income Homes	14	4,135,857	Urban	Black	No	No	Yes	No
